# COVID-19 in medium-sized municipalities in the 14 health macro-regions of Minas Gerais, Brazil

**DOI:** 10.1590/1414-431X2021e11191

**Published:** 2021-08-20

**Authors:** W. de Paula-Júnior, R.C.R.M. do Nascimento, R.S. Matiles, F.F. de Lima-Neto, M.C.R. Leles, H.N. Guimarães, A. Grabe-Guimarães

**Affiliations:** 1 Universidade Estadual de Montes Claros, Montes Claros, MG, Brasil; 2 Programa de Pós-graduação em Ciências Farmacêuticas, Escola de Farmácia, Universidade Federal de Ouro Preto, Ouro Preto, MG, Brasil; 3 Faculdade de Ciências Gerenciais, Manhuaçu, MG, Brasil; 4 Universidade Federal de São João Del-Rei, Campus Alto Paraopeba, Ouro Branco, MG, Brasil; 5 Escola de Engenharia, Universidade Federal de Minas Gerais, Belo Horizonte, MG, Brasil

**Keywords:** SARS-CoV-2, Epidemiology, Prevalence, ICU beds, Pandemic, Time series analysis

## Abstract

The present study focused on the scenario of confirmed cases of SARS-CoV-2 infection in the state of Minas Gerais (MG), Brazil, from March 2020 to March 2021. We evaluated the evolution of COVID-19 prevalence and death in one municipality from each of the 14 health macro-regions of MG state. Socio-demographic characteristics and variables related to the municipalities were analyzed. The raw dataset used in this study was freely sourced from the website Brasil.io. From the raw dataset, two time series were extracted: the cumulative confirmed cases of COVID-19 and cumulative death counts, and they were compared to the state data using a nowcasting approach. In order to make time series comparisons possible, all data was normalized per 100,000 inhabitants. When analyzing in light of colored wave code interventions initiated in August 2020 in MG, for the majority of the municipalities, there was an absence of clear influence on prevalence and deaths. The national holidays in the first semester of 2020 had a small impact on the COVID-19 prevalence of the municipalities, but the holidays in the second semester of 2020 and beginning of 2021 caused important impacts on COVID-19 prevalence. The low number of ICU beds in some municipalities contributed to the higher number of deaths. The analysis showed here is expected to contribute to the improvement of decision making of the MG government, as it opened a huge possibility to have the total macro-regions and state data analyzed.

## Introduction

COVID-19 (coronavirus disease 2019) is a multifactorial public health problem that requires adequate epidemiological surveillance (1,2). Strategic plans to respond to the SARS-CoV-2 (severe acute respiratory syndrome - coronavirus 2) infection, especially to contain its spread and reduce morbidity and mortality, have been incorporated into the government agendas of most countries. The worldwide mobilization began on January 30, 2020, when the World Health Organization (WHO) officially recognized the COVID-19 pandemic as an international public health emergency (3).

The new coronavirus arrived in Latin America from different locations of North America, Asia, and Europe (4). The first case of COVID-19 confirmed in Brazil was in São Paulo, in February 2020. Since then, there has been an increasing incidence and number of deaths from this cause (5). Brazil has social and economic disparities among its regions, with inequalities related to coping with COVID-19, such as availability of diagnostic tests, number of intensive care unit (ICU) beds, access to and understanding of disease information, and political decisions in controlling the pandemic (6).

There have been several epidemiological studies evaluating the potential factors influencing the spread of COVID-19 infection and number of deaths in Brazil, all aimed at helping the Brazilian authorities to make critical decisions and direct new strategies for controlling the COVID-19 pandemic (7-9). For example, there was a detailed analysis of the spatial dispersion of COVID-19 in São Paulo state, Brazil, aimed at providing real-time responses (10). Another analysis of the epidemic evolution of COVID-19 in the Regional Health Departments of São Paulo state showed that none of the departments achieved the green phase of the sanitary plan, meaning flexible social distancing, during the first 200 days of the pandemic, regarding new confirmed cases of COVID-19, and new notifications of severe acute cases (11). The use of an applied technology in the spatial identification of transmission, prevention, and control of the SARS-CoV-2 infection was developed in Fortaleza, capital of Ceará Sate in Brazil, with the purpose of supporting the decision-making process of the Health Department of Ceará, during the COVID-19 outbreak (12). The true association between economic inequality and infection and death by COVID-19 in Brazil was shown, taking the wide social, geographic, and economic diversity as an important factor for pandemic impact (1).

Minas Gerais state (MG), located in the Southeast region of Brazil, is the state with the largest number of municipalities, corresponding to 15.3% (n=853) of the Brazilian municipalities. MG is also the pioneer state in the implementation of health regionalization, establishing a territorial and population basis for projecting needs, prioritizing the allocation of financial resources, and management decentralization (13). The challenges of facing the COVID-19 pandemic in MG have been similar to the national ones, due to its large area and socio-economic disparities among its regions.

In May 2020, safeguarding the autonomy of each municipality, and considering the epidemiological aspects of COVID-19 together with the economic demands, the MG government created the “*Minas Consciente - Retomando a Economia do Jeito Certo*” plan (translated as “Conscious Minas - Restoring the economy in the right way”, and referenced hereinafter simply as “Conscious Minas Plan”) (14). The first version of this plan color-coded the need and rigidity of interventions to control virus transmission with three colors waves (using the metaphor of a traffic light colors: green, yellow, and red), where green means mild interventions and red means tighter restrictions. The plan included a health protocol that gathers guidelines for employers, workers, tourists, and the general population on appropriate practices to control the spread of COVID-19. Some indicators were adopted by health macro-regions and micro-regions for this analysis, such as COVID-19 incidence rate, occupancy of adult ICU beds, and number of hospital beds per 100,000 inhabitants. In March 2021, the color wave code was revised to include purple, with tougher restriction rules, as an attempt to contain increasing COVID-19 numbers (15).

The present study focused on the scenario of confirmed cases of SARS-CoV-2 infection in MG from March 2020 to March 2021. We evaluated the evolution of COVID-19 prevalence and deaths in one municipality from each of the 14 health macro-regions of MG (13). Analyzing the epidemiological profile of COVID-19 in different geographic regions of the state can contribute to a better understanding of the pandemic's evolution, supporting the adoption of strategies to mitigate the morbidity and mortality associated with this disease and consequently to minimize its post-pandemic effects. The surveillance data from each municipality was also compared with data from all of MG. A non-parametric comparison was created, which we called signed squared differences (SSD). This approach considered the state time series as a trend, classifying municipalities as above or below the state trend. The goal of this study was also to assess the existence of causality between the holidays and surveillance data, as well as to monitor the impact of color wave code interventions on reduction of prevalence and deaths.

## Material and Methods

We performed a time series analysis of reported cases of COVID-19 in MG, corresponding to an ecological study. The data obtained from March 8, 2020 to March 27, 2021 were analyzed, the times corresponding to the 11th epidemiological week of 2020 to the 12th of 2021 (16,17).

Public information with open access was used, which does not allow access to individual identification, following the criteria of Resolution No. 510 of 2016, of the National Council of Ethics in Research (18).

**Figure 1:**
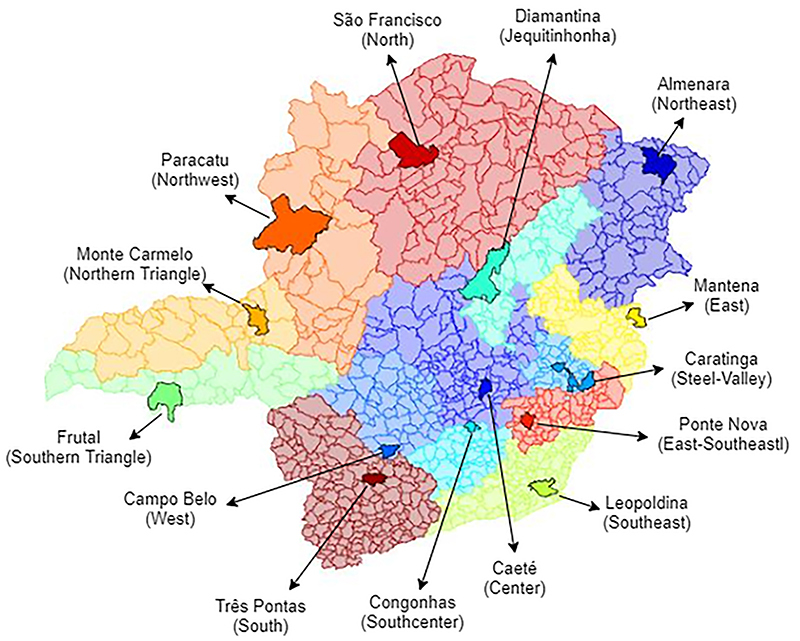
The 14 health macro-regions of Minas Gerais (MG) state in Brazil, according to the Master Plan of MG (13). The 14 municipalities evaluated are highlighted in each macro-region.

To analyze the epidemiological profile of COVID-19 in municipalities in different parts of MG, one municipality was selected from each state health macro-region, considering the Regional Health Master Plan (RDP) (13). Fourteen mid-sized municipalities in the respective 14 health macro-regions of MG were chosen for the study, and their geographic locations are shown in Figure 1. The adopted selection criteria were to be a medium-sized municipality with a population between 25 and 100 thousand inhabitants and to have at least one reported case of COVID-19 up to May 15, 2020.

The total population of each municipality was obtained from the database of the Brazilian Institute of Geography and Statistics (IBGE) (19), estimated for 2019, and used to normalize data per 100,000 inhabitants. Also sourced from IBGE, all the data for sociodemographic characteristics and variables related to the municipalities were analyzed: demographic density, age group, sex distribution, education level, gross domestic product (GDP, in local currency, R$), and human development index (HDI). This last index involves the educational, health, and *per capita* income metrics, normalized to range from 0 to 1. The percentage of the population over 50 years old was used because it was considered a major risk group. The education level of the population for each municipality was described as the percentage of illiterate plus those with incomplete primary school.

The total number of ICU beds for COVID-19 patient treatment was obtained from the database of the Department of Information Technology of the Unified Health System (DATASUS), by the National Registry of Health Establishments (CNES) (20), and it was normalized to 100,000 inhabitants.

The raw dataset used in this study was freely sourced from the website Brasil.io (21) in .csv format. The data were compiled daily and organized from the official epidemiological bulletins of MG state. Two time series were extracted from the raw dataset: the cumulative confirmed cases of COVID-19 and cumulative death counts.

**Figure 2:**
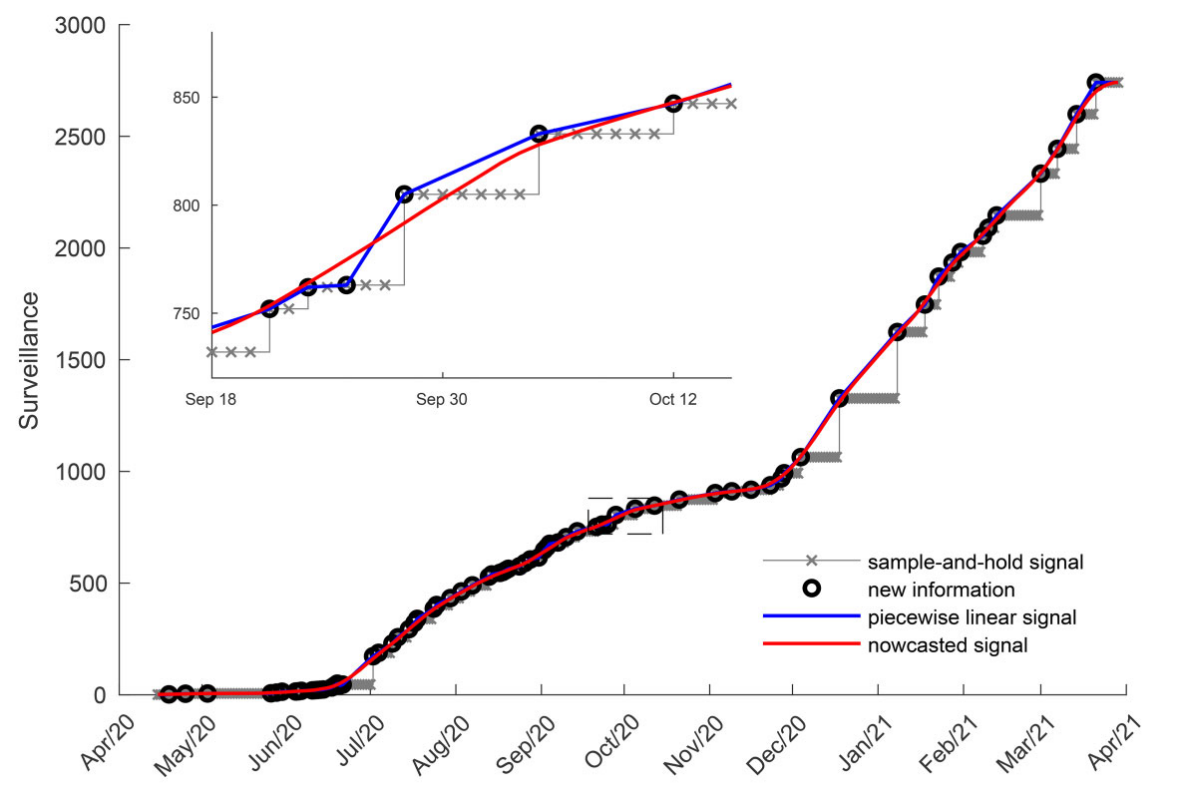
Details of the pre-processing approach for the raw COVID-19 surveillance data. The original database signal interpolated by the sample-and-hold technique is shown in a staircase-shaped discrete-time signal, where each X-mark means a daily sample. With each new information arrival (marked with O) a new step is formed. In blue is the piecewise linear signal, formed by line segments connecting the unevenly time series samples. From resampling and smoothing with moving average digital filter the piecewise linear signal, the nowcasted signal was derived, shown in red. The nowcasted signal does not necessarily cut through the information arrival points (O).

The COVID-19 surveillance data were unevenly sampled due to difficulties in collecting information resulting in reporting delays. To mitigate this artifact, the raw time series was resampled daily, by the data source, using a sample-and-hold approach. The data were held until the incoming of new data, resulting in a staircase-shaped discrete-time signal. It was observed that some municipalities showed long delays of up to two weeks in case reports, so that the sample-and-hold approach resulted in an exacerbated staircase shape. Therefore, in this study, a nowcasting approach (the art of “predicting the present”) was employed. Line segments were used to connect the uneven time series samples, resulting in a piecewise linear signal. This signal was then resampled daily and smoothed with a 7-day moving average digital filter (Figure 2).

The other time series was derived from the nowcasted COVID-19 surveillance time series, cumulative confirmed cases, and death counts. These time series were generated for each municipality, each heath macro-region, and all MG. To make time series comparisons possible, all of them were normalized per 100,000 inhabitants. Except when explicitly mentioned, all surveillance data in this study were per 100,000 inhabitants and referred to as COVID-19 prevalence and deaths. Lethality was the percent of deaths from the prevalence.

**Figure 3:**
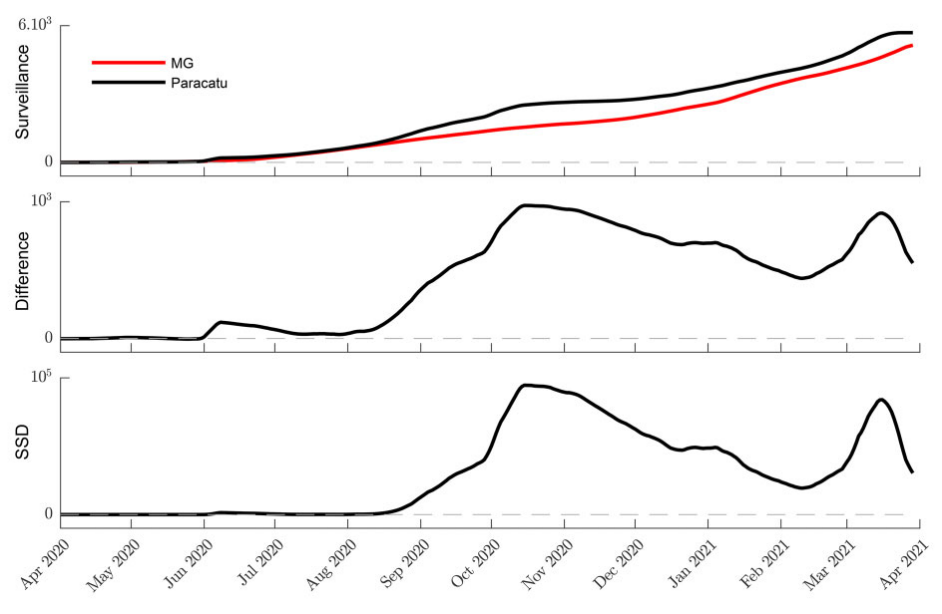
Details of signed squared difference (SSD) time series construction. The SSD was developed to show the evolution of municipality data in relation to the state, considered as a reference curve. The figure shows both the difference and the SSD time series. The difference signal can be considered a detrended time series (in relation to state time series). The SSD time series was used to spotlight when the surveillance data of one municipality deviated from the reference.

Considering the MG state time series as the main trend, a metric was derived to observe how the municipalities deviated from this trend. These deviations were calculated by the signed squared difference (SSD) between the daily samples of the municipality and the state time series (Figure 3). Being x_*t*_ and y_*t*_ the nowcasted values for municipality and state, respectively, at time *t*, the SSD_*t*_ was calculated by: SSD_*t*_ = sign(x_*t*_–y_*t*_) × (x*_t_*–y*_t_*)^2^.

The SSD time series represents a detrend municipality signal augmented by the square operation. A growing positive SDD is an indicator requiring attention to the respective municipality.

To see if there was a causality between health interventions and turning points in the time series, as well as to evaluate the impact of these interventions, colored bands representing the health color wave code and lines indicating the main holidays in Brazil were superimposed on the timelines. The holidays with potential influence on prevalence evaluated were June 11, (Corpus Christi, a religious holiday), September 7 (Brazilian Independence Day), October 12 (Nossa Senhora Aparecida, a religious holiday), November 15 (Republic Proclamation and municipalities national election), December 25 (Christmas), January 1 (New Year’s Day), and February 16 (Carnival).

All the analyses were performed using MATLAB R2018a (The MathWorks, Inc., USA).

## Results

MG registered its first case of COVID-19 on March 8, 2020 in the 11th epidemiological week. After a year of pandemic, in the 12th epidemiological week of 2021, MG had 1,093,539 confirmed COVID-19 cases and 23,366 deaths.

The sociodemographic characteristics of the municipalities are presented in Table 1. All the municipalities included in this study were medium-sized, with populations ranging from 27,664 (Mantena) to 93,158 (Paracatu), and heterogeneous in population density. All 14 municipalities had a homogeneous distribution of sex, with females making up 50.76% (SD=0.97) of the population. Regarding education, all municipalities had a predominance of illiterate or incomplete primary level education (56.21%; SD=5.25). Almenara, São Francisco, and Mantena had the highest rates of low schooling. São Francisco also had the lowest economic indicators (GDP and HDI).

**Table 1:** Sociodemographic characteristics of municipalities from the 14 health macro-regions of Minas Gerais state.

Municipalities	Population	Inhabitants/km^2^	Female (%)	Age>50 years (%)	NE/PES (%)	GDP per capita (R$)	HDI	ICU beds/100,000 inhabitants
Almenara	41,896	16.90	49.6	22.18	65.9	11,873	0.642	23.86
Caeté	45,047	75.11	51.3	22.32	50.1	13,021	0.728	-
Campo Belo	54,186	97.58	51.2	27.28	58.9	18,066	0.711	18.50
Caratinga	92,062	67.72	51.1	22.40	58.4	17,516	0.706	114.05
Congonhas	54,742	159.57	50.9	20.50	48.2	30,573	0.753	18.26
Diamnatina	47,723	11.79	51.5	19.63	53.4	15,046	0.716	52.38
Frutal	59,496	22.03	49.4	23.06	54.4	15,046	0.730	-
Leopoldina	52,587	54.22	52.0	26.54	53.3	19,468	0.726	36.13
Mantena	27,664	39.57	51.7	24.85	62.2	13,394	0.675	18.07
Monte Carmelo	47,809	34.08	49.6	20.04	58.5	22,628	0.728	41.83
Paracatu	93,158	10.29	49.9	16.41	49.4	38,000	0.744	5.36
Ponte Nova	59,742	121.94	52.1	24.17	54.2	27,330	0.717	48.54
São Francisco	56,323	16.27	49.3	18.50	64.4	8,713	0.638	-
Três Pontas	56,746	78.08	50.4	22.39	55.7	21,971	0.731	17.6

NE: no education; PES: partial elementary school; GDP: gross domestic product; HDI: human development index; ICU: intensive care unit. Data source: Brazilian Institute of Geography and Statistics (IBGE, 2021) and National Health Facilities Register (DATASUS, 2021).

Great variability was observed in the distribution of ICU beds in municipalities (Table 1). Caeté, Frutal, and São Francisco had no ICU beds, essential for the management of patients with more severe COVID-19. The municipalities with the highest number of ICU beds per 100,000 inhabitants were Caratinga, Diamantina, and Ponte Nova.

**Figure 4:**
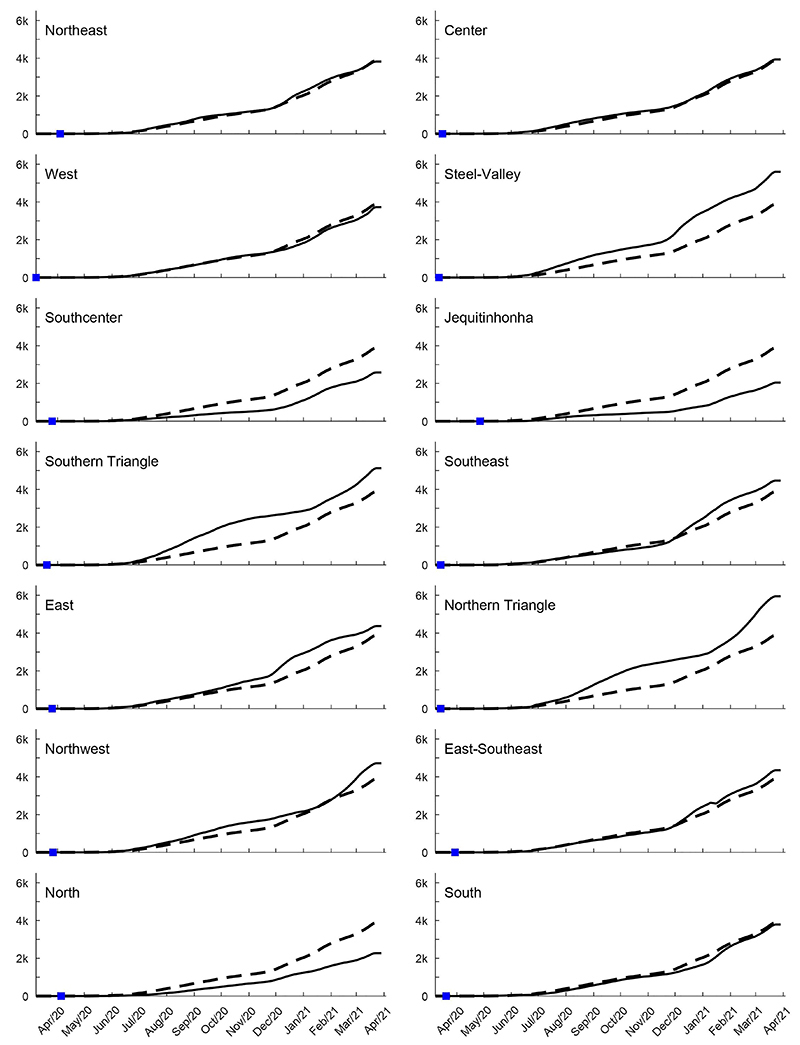
COVID-19 prevalence (per 100k inhabitants) for the 14 Minas Gerais macro-regions (full lines) contrasted with the whole state (segmented lines). The first confirmed case within each macro-regions is shown with a blue square mark.

The analysis of COVID-19 prevalence for each macro-region compared to MG is shown in Figure 4. Compared to MG prevalence (5045.67 cases/100,000 inhabitants) on March 27, 2021, the macro-regions of Northeast, West, Center, East-Southeast, and South had a similar profile. The macro-regions of Southcenter, North, and Jequitinhonha had lower prevalence of COVID-19 most of the time compared to the state. The macro-regions of Steel-Valley, Southern Triangle, and Northern Triangle and East had higher prevalence compared to the state most of the time. The Southeast had a similar prevalence profile until December 2020 and after that had a higher prevalence. The Northeast had a similar prevalence to the state until August 2020 and in January and February 2021, but a higher prevalence in the other months.

**Figure 5:**
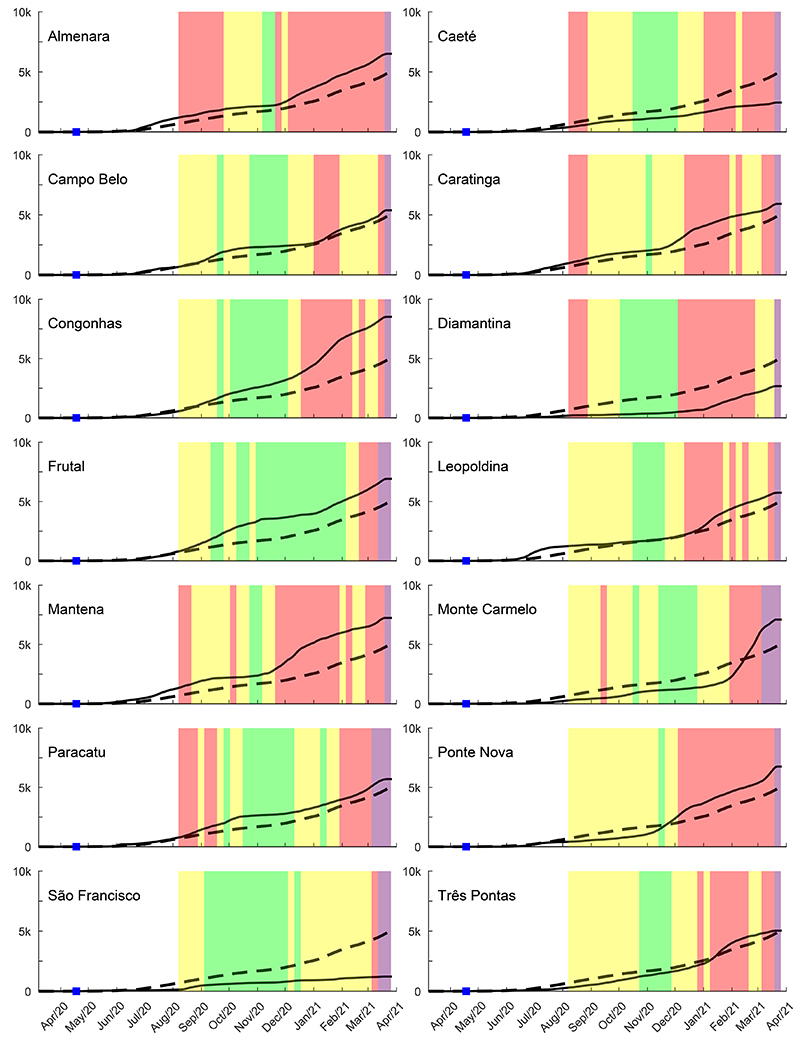
COVID-19 prevalence (per 100k inhabitants) for the 14 municipalities of Minas Gerais (MG) state macro-regions (full lines) contrasted with the whole MG state (segmented lines). The colored bands from August 2020 to March 2021 represent the color wave codes defined by the Conscious Minas Plan. The first confirmed case within each macro-regions is shown as a blue square mark.

**Figure 6:**
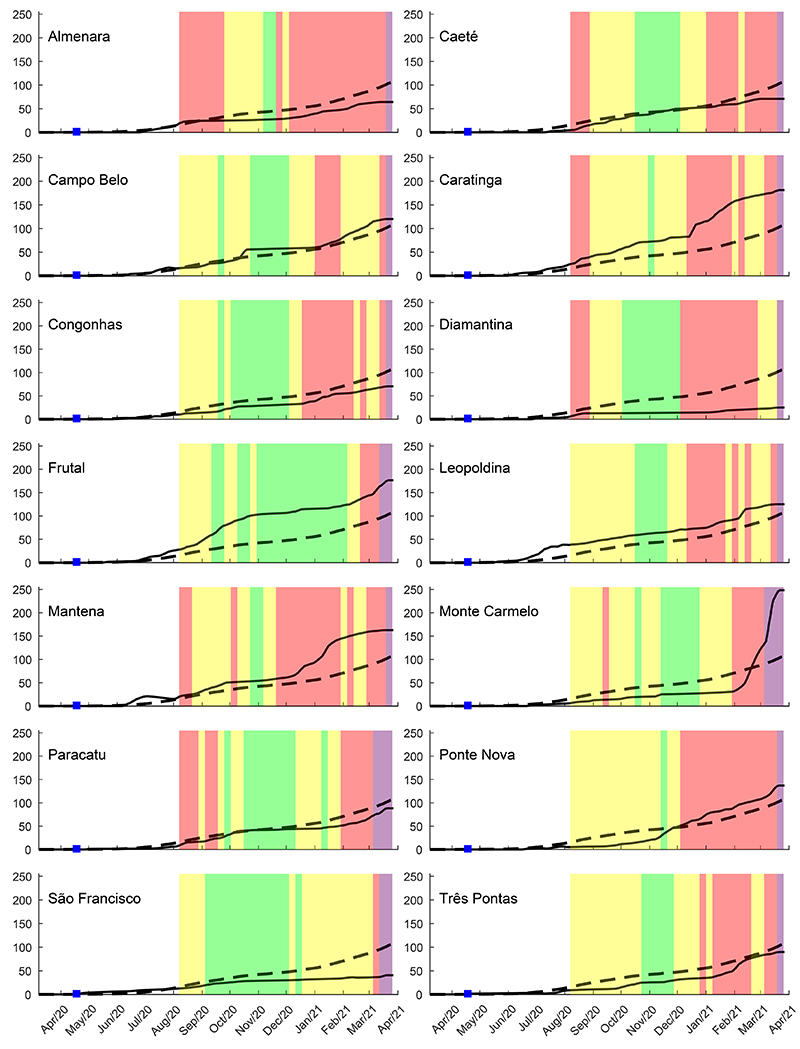
Deaths resulting from COVID-19 (per 100k inhabitants) for the 14 municipalities of Minas Gerais (MG) state macro-regions (full lines) compared with the whole MG state (segmented lines). The colored bands from August 2020 to March 2021 represent the color wave codes defined by the Conscious Minas Plan. The first confirmed case within each macro-region is shown as a blue square mark.

The reported cases of COVID-19 and confirmed deaths in each municipality per 100,000 inhabitants were followed for 54 weeks, or 385 days (Figures 5 and 6). We compared COVID-19 cases reported by the municipalities to the cases of all MG, to qualitatively describe trends. In the first three months of the pandemic (from the 11th to the 24th epidemiological weeks) for these 14 municipalities, the majority was similar to the state. Paracatu (206), Mantena (195), and Leopoldina (106) had the highest numbers of COVID-19 cases. Frutal (31), Diamantina (20), and Monte Carmelo (16) had the lowest numbers. The number of confirmed deaths was highest in Caratinga (4) and São Francisco (3) in the first three months.

The first case reported for each municipality is shown in Figure 4 by a blue square mark. Campo Belo was the first one among the 14 municipalities to report the first case, in the 13th epidemiological week. Monte Carmelo, Frutal, and Paracatu had the first case notified in the same week of April 2020 (Figure 5). Ponte Nova, Mantena, and Caratinga had the first case later, in the 19th epidemiological week, in May 2020. The occurrence of the first COVID-19 case in Congonhas and Diamantina was in the 20th epidemiological week of 2020 (Figure 5).

Although all municipalities had an increasing profile both for COVID-19 prevalence and deaths (Figures 5 and 6), when compared to the state numbers along the 54 weeks evaluated, nine municipalities had a higher prevalence (Almenara, Campo Belo, Caratinga, Congonhas, Frutal, Leopoldina, Mantena, Paracatu, and Ponte Nova) most of the time. Five municipalities maintained a lower number of cases most of the time (Caeté, Diamantina, Monte Carmelo, São Francisco, and Três Pontas) compared to the state prevalence. Monte Carmelo had lower prevalence until January 2021, but in February, it had a huge increase, and Três Pontas had a similar profile (Figure 5).

Color wave code interventions (14) were initiated in August 2020 in MG, and it was expected to influence prevalence at least 15 to 30 days after the adopted restrictions in each municipality. The colors green (the most flexible), yellow, red, and purple (the tightest) of “Conscious Minas Plan” (14,15) of the state government are also shown in Figure 5 for each municipality studied to analyze the impact of the plan on COVID-19 cases and number of confirmed deaths. Also at each municipality, local governments published decrees regulating the Plan based on the local economic and epidemiological situation. When analyzing in light of the color wave code, we observed, for the majority of the municipalities, an absence of clear influence on prevalence increase or decrease and also for deaths. Congonhas and Frutal had green and yellow waves most of the time, and their prevalence (Figure 5) was higher, compared to the state. Almenara used the red wave rules most of the time and it was not enough to prevent the prevalence increase compared to the state (Figure 5). Caeté, Diamantina, Monte Carmelo, São Francisco, and Três Pontas maintained a lower prevalence compared to the state, despite the use of green and yellow wave rules most of the time in 2020.

When analyzing death due to COVID-19 (Figure 6), the profile of ten municipalities was similar to the COVID-19 infection prevalence; for three other municipalities the death profile was better, meaning less deaths despite the increasing number of new cases. We also show the color wave code of the “Conscious Minas Plan” in Figure 6.

Almenara, Congonhas, Diamantina, Paracatu, São Francisco, and Três Pontas had fewer deaths compared to MG all along the time evaluated (Figure 5). The municipalities with the highest lethality, also compared to the state (2.14%) at the end of the 385 days evaluated, were Caratinga (3.19%), Caeté (3.12%), Frutal (2.51%), Campo Belo (2.41%), Mantena (2.34%), and Leopoldina (2.25%). The lowest lethality occurred in Congonhas (0.83%), Diamantina (0.97%), and Almenara (1.06%); also, deaths were lower when compared to MG over the evaluation time (Figure 6). São Francisco, the municipality that used the red wave restriction rules the least, also had a high lethality (2.99%), despite the low COVID-19 prevalence and few deaths (Figures 5 and 6) compared to the COVID-19 prevalence and deaths of the state. Three municipalities, Caeté, Frutal, and São Francisco, did not have ICU beds (Table 1), and they are among the ones with the highest lethality. Monte Carmelo (2.29%) had fewer deaths compared to the state until February 2021, followed by a huge increase afterwards (Figure 4). Ponte Nova (1.99%) surpassed the state deaths since December 2020. Paracatu (lethality=1.45%) and Campo Belo had a similar profile of deaths compared to the state (Figure 5).

**Figure 7:**
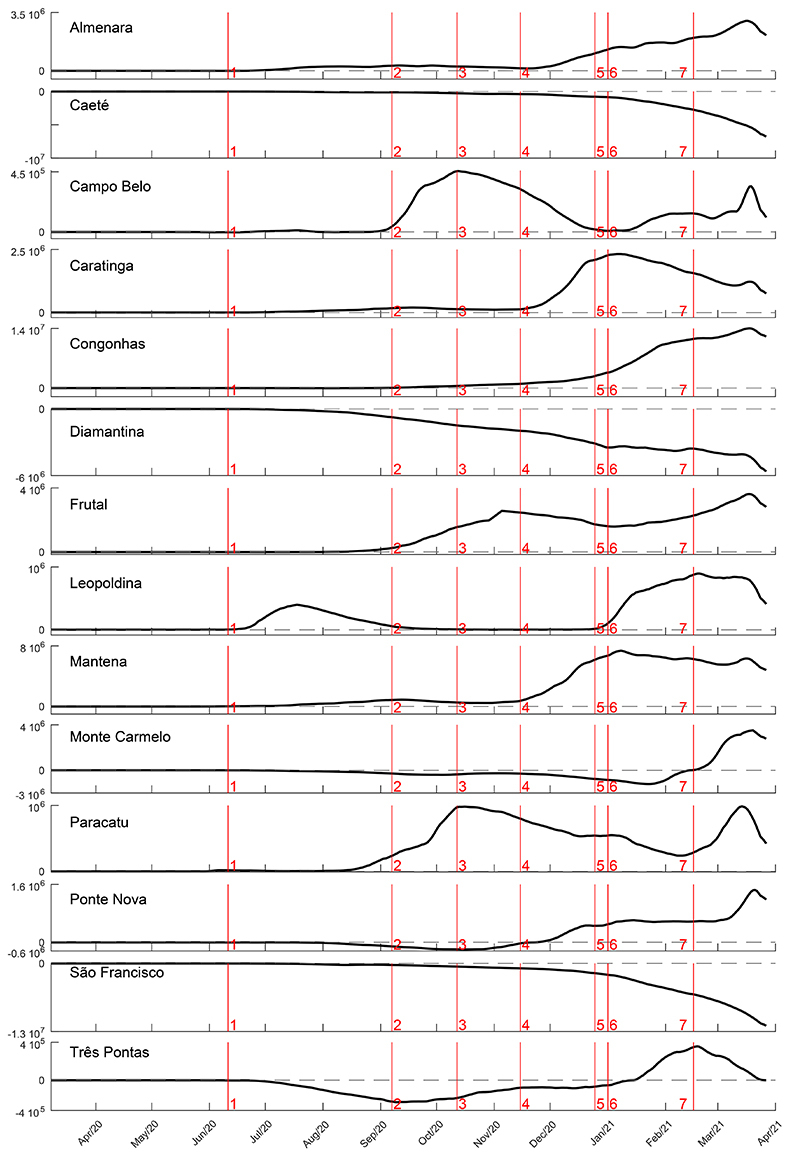
COVID-19 signed squared difference (SSD) time series for the 14 municipalities of Minas Gerais state macro-regions. The red vertical lines indicate the main Brazilian holidays: 1: Corpus Christi ; 2: Brazilian Independence Day; 3: Nossa Senhora Aparecida; 4: Republic Proclamation and municipalities national election; 5: Christmas; 6: New Year’s Day; 7: Carnival.

The COVID-19 prevalence of the municipalities contrasted that of MG (Figure 7). The mathematical treatment given to the data amplified the turning points detected along the time evaluated, and we also included the most important holidays in the state to describe their potential influence on new COVID-19 cases in the 14 municipalities. The first holiday as a potential factor to increase the prevalance of COVID-19 was June 11th (Corpus Christi) in Almenara and Leopoldina. Campo Belo, Congonhas, Frutal, Paracatu, and Três Pontas showed increasing confirmed cases around September 7th and October 12th, probably influenced by the increase in prevalence in Frutal, Monte Carmelo, and Ponte Nova; however, a decrease in prevalence was observed in Campo Belo and Paracatu during the same period. From November 15, 2020, the day of municipal elections in the whole country, four municipalities had a huge increase in prevalence: Almenara, Caratinga, Mantena, and Ponte Nova. In addition, Christmas holiday and New Year celebrations marked the prevalence increase in Almenara, Campo Belo, Congonhas, Frutal, Leopoldina, Ponte Nova, and Três Pontas. Caeté, Diamantina, and São Francisco showed a decrease in COVID-19 prevalence compared to the state. Carnival seemed to cause an increase in prevalence compared to the state in Almenara, Congonhas, Frutal, Monte Carmelo, and Paracatu (Figure 7). Thus, most of municipalities showed oscillations in confirmed cases regarding the holidays and to the color wave code of the “Conscious Minas Plan” (14,15).

## Discussion

MG has an area of 586,528 km^2^ and a diversity of climate, vegetation, culture, and economy (19). The COVID-19 prevalence and deaths were analyzed for 54 epidemiological weeks, from 2020 to 2021, a period to understand the characteristics of the COVID-19 pandemic. The data from 14 municipalities were analyzed, each from one of the 14 macro-regions defined by PDR/MG (13). The diversity of COVID-19 prevalence and death led us to some considerations regarding sociodemographic data and differences between the municipalities.

The probability of transmission of SARS-CoV-2 is higher in gatherings of people, and therefore, municipalities with a higher population density are expected to have a greater spread of the disease (22). However, the results did not indicate this trend in the 14 municipalities analyzed. Congonhas has a higher population density and a high COVID-19 prevalence, and Diamantina has a lower population density and a low prevalence compared to the state, as expected; however, Paracatu has the lowest population density but a higher prevalence compared to the state. These observations of demographic density showed an absence of association as an isolated factor. Strategies used to contain the disease spread by reducing gatherings and adopting prophylaxis habits (23) may have contributed to the observed differences, despite the adoption of the restrictions given by the color wave code of the “Conscious Minas Plan” (14,15).

An American study, based on the modeling of 48 possible social distancing scenarios, indicated stepping-down as the best strategy. This consists of starting at specific social distancing rules and then dividing by half for the next 3 time-windows, and on the fifth time-window social distancing is back to its initial value, and the process is repeated (24). This idea could represent, for example, rules of red wave adopted for one week, then rules of green/yellow for 3 weeks, then again red wave, using the definitions of the MG state plan (14). Perhaps if the same restriction rules were adopted for all the municipalities at the same time as proposed by Kennedy et al. (24), there would have been better advances in pandemic control. All the color wave code restrictions of the 14 municipalities included social isolation “when possible”, but there was no measure of the population behavior regarding this parameter.

The national holidays in the first semester of 2020 had little impact on the COVID-19 prevalence of the municipalities, leading us to think about behavior in the face of the uncertain and unsafe new situation of the pandemic. Although the majority of holidays likely extend into the weekend, November 15 was a Sunday, and it was the one with higher prevalence increase soon after it or up to 15 days after. This observation could be explained by the national elections in all municipalities, probably a situation with many gatherings. Christmas and New Year holidays are naturally the ones when people have more family events and all kinds of gatherings, which contributed to the coronavirus spread. A narrative review evaluating prevalence of asymptomatic SARS-CoV-2 infection, including 15 studies around the world, showed a range of 30 to 96% (25) and indicated social distancing as a key step for a pandemic solution.

To organize the provision of health services at three levels of care, according to the low, medium, and high complexity, MG was divided into 14 macro-regions and 89 health micro-regions (13). Some municipalities evaluated are important from an economic and health standpoint compared to other municipalities in their respective macro-region. Paracatu, for example, is an important mining hub in its region, and it has a highway connecting MG to the Central-West region of the country (26). Another study in São Paulo based on COVID-19 surveillance data evidenced the main routes of disease dispersion from capital to inner state (27). The municipalities evaluated in this study are in three categories of assistance: Almenara, Caeté, Campo Belo, Caratinga, Congonhas, Frutal, Leopoldina, Mantena, Montes Carmelo, and Três Pontas are central hub municipalities of their micro-regions but not of their macro-regions. Diamantina and Ponte Nova are central hubs of their micro- and macro-regions. Paracatu and São Francisco are not central hubs. Additionally, all the municipalities where the first cases occurred among the 14 analyzed are in land traffic routes between MG and other regions of Brazil, such as Frutal, Monte Carmelo, Paracatu, Ponte Nova, Caratinga, and Mantena.

Among the municipalities included in this study, three do not have ICU beds, requiring the referral of cases for hospitalization in another city. Four municipalities have an unsatisfactory number and fewer ICU beds than the national average (15.6 per 100,000 inhabitants). Although the number of ICU beds in Brazil is within the WHO recommendation (10-30 ICU beds per 100,000 inhabitants) (28), its distribution among the regions is not homogenous and is mostly concentrated in state capitals, reducing the effectiveness of health care in the state inner municipalities (29). The three municipalities without ICU beds were the ones with the highest lethality. One of them was Caeté, a municipality very close to the capital of MG, Belo Horizonte, which explains the absence of ICU beds but not the higher lethality. Frutal is near other municipalities with hospitals with COVID-19 beds, but on the other hand, São Francisco, the municipality with a high lethality, is located far from municipalities with adequate health care. São Francisco also had the highest number of deaths in the first three months and did not have any ICU beds until the end of this analysis period. The data shown here confirm the poor distribution of ICU beds and the precariousness of the conditions of care for critically ill patients with COVID-19. Increased deaths can be related to greater shortage of ICU beds and pulmonary ventilators (30). If we consider that ICU hospitalized patients have at least a 37% chance of cure, as shown by a national data analysis of the first four months of the pandemic in Brazil (31), ICU absence is then a key factor impacting deaths. The crisis generated by the pandemic highlighted the weaknesses of the health systems in the world, showing the lack of preparation for such an emergency, and drastically affected the provision of services and health care (32). Its implications will remain for a long time, requiring efforts from society, government, specialists, and health professionals to ensure efficient recovery (33). The health RDP was updated in 2019, and before the pandemic of the new coronavirus. It gained a new update in October 2020 (13), as an attempt to contribute to new strategies to postpone the pandemic; but the impact of this action needs time to become a reality.

Considering the age above 50 years as posing the highest risk of morbidity and mortality due to COVID-19 (34), it was observed that the municipalities with the highest percentage of inhabitants in this age group (Table 1) were Campo Belo, Leopoldina, Mantena, and Ponte Nova. Paracatu had the smallest population in this age group, which suggests less possibility of COVID-19 cases, but the opposite was observed. Although all age groups are equally susceptible to contracting the new coronavirus, mortality in individuals over 59 years of age has been shown to be higher in Brazil (35,36). A descriptive cross-sectional study based on secondary data between January 1 and August 20, 2020 in Rondônia, in North Brazil, analyzed 49,804 confirmed COVID-19 cases and showed a greater occurrence of deaths in older age groups and among males (37).

A study carried out with the objective of estimating the proportion of adult individuals at risk for COVID-19 in Brazil showed that, although no differences were found between the sexes, the risk factors were twice as high in adults with lower levels of education, compared to those with more than high school (38). This factor certainly contributes to a lower capacity to understand and adopt preventive health habits, even if they are determined through legal documents of the municipalities. Another important aspect is the possibility that the health system may collapse and not be able to meet the high demand for new patients, one of the main reasons for the recommended social isolation (30).

With regard to GDP, an indicator of the flow of final goods and services produced in a period, Paracatu, Congonhas, Ponte Nova, Monte Carmelo, and Três Pontas showed the highest values, and a connection to the prevalence and deaths was not observed. São Francisco, with the lowest GDP, had the highest lethality although one of the lowest prevalence. All HDI values of the municipalities were in the range of human average, between 0.500 and 0.799, similar to the national value of 0.761 and 0.731 for MG. The lowest HDI was found for Almenara and São Francisco, located in the Northeast and North macro-regions, respectively, considered the most unreliable in MG. According to a study carried out in Brazil, the progression in the incidence and mortality rates due to COVID-19 was more prominent in states with greater socio-economic inequalities, while among the states with lower inequality, there were modest increases in these epidemiological rates (1); 59.8% of variation in the incidence of COVID-19, was explained by income inequality, substantial home gathering, and higher mortality, indicating that socioeconomic factors influenced the evolution and impact of COVID-19 in Brazil (2). However, another study evaluated COVID-19 prevalence in relation to HDI, and the authors did not show greater prevalence of the disease in municipalities with precarious conditions (39).

This study, conducted with data from 14 municipalities, may contribute to a better understanding of the challenges faced by MG during the pandemic and to reinforce some findings from numerous other epidemiological studies undertaken in the most diverse regions of Brazil and worldwide. The main result observed here as a potential factor influencing COVID-19 prevalence was the holidays in some municipalities, where the numbers increased substantially. The restriction rules guided by the color wave code of the MG government (14) should have taken into account the factors with positive impact in some municipalities to be employed in the other ones. In addition, the analysis presented here is expected to contribute to improving the decision-making of the MG government, as it opened a huge possibility to have the total macro-regions and state data analyzed.

The results from the analyzed data of the municipalities of MG, a central state in Brazil, indicated the importance of planning the restrictions given by the color wave code, social distancing reinforcement especially during the holidays, the importance of more ICU beds especially in those municipalities without them, and the importance of surveillance as a strong tool to predict the risks.
